# The impact of pesticide suicide on the geographic distribution of suicide in Taiwan: a spatial analysis

**DOI:** 10.1186/1471-2458-12-260

**Published:** 2012-04-02

**Authors:** Shu-Sen Chang, Tsung-Hsueh Lu, Jonathan AC Sterne, Michael Eddleston, Jin-Jia Lin, David Gunnell

**Affiliations:** 1 School of Social and Community Medicine, University of Bristol, Bristol, UK; 2 Centre for Suicide Research and Prevention, The University of Hong Kong, Hong Kong SAR, China; 3 Ju Shan Hospital, Taoyuan, Taiwan; 4 Institute of Public Health, College of Medicine, National Cheng Kung University, Tainan, Taiwan; 5 Clinical Pharmacology Unit, University/BHF Centre for Cardiovascular Science, University of Edinburgh, Edinburgh, UK; 6 Department of Psychiatry, Chi-Mei Medical Center, Tainan, Taiwan; 7 Department of Psychiatry, School of Medicine, College of Medicine, Taipei Medical University, Taipei, Taiwan; 8 Institute of Public Health, College of Medicine, National Cheng Kung University, No.1, Daxue Rd., East District, Tainan City 70101, Taiwan

**Keywords:** Suicide, Pesticide, Mapping, Ecological studies, Taiwan

## Abstract

**Background:**

Pesticide self-poisoning is the most commonly used suicide method worldwide, but few studies have investigated the national epidemiology of pesticide suicide in countries where it is a major public health problem. This study aims to investigate geographic variations in pesticide suicide and their impact on the spatial distribution of suicide in Taiwan.

**Methods:**

Smoothed standardized mortality ratios for pesticide suicide (2002-2009) were mapped across Taiwan's 358 districts (median population aged 15 or above = 27 000), and their associations with the size of agricultural workforce were investigated using Bayesian hierarchical models.

**Results:**

In 2002-2009 pesticide poisoning was the third most common suicide method in Taiwan, accounting for 13.6% (4913/36 110) of all suicides. Rates were higher in agricultural East and Central Taiwan and lower in major cities. Almost half (47%) of all pesticide suicides occurred in areas where only 13% of Taiwan's population lived. The geographic distribution of overall suicides was more similar to that of pesticide suicides than non-pesticide suicides. Rural-urban differences in suicide were mostly due to pesticide suicide. Areas where a higher proportion of people worked in agriculture showed higher pesticide suicide rates (adjusted rate ratio [ARR] per standard deviation increase in the proportion of agricultural workers = 1.58, 95% Credible Interval [CrI] 1.44-1.74) and overall suicide rates (ARR = 1.06, 95% CrI 1.03-1.10) but lower non-pesticide suicide rates (ARR = 0.91, 95% CrI 0.87-0.95).

**Conclusion:**

Easy access to pesticides appears to influence the geographic distribution of suicide in Taiwan, highlighting the potential benefits of targeted prevention strategies such as restricting access to highly toxic pesticides.

## Background

Pesticide self-poisoning is one of the most commonly used suicide methods worldwide, accounting for 250 000-370 000 deaths every year - around one third of the world's suicides [1]. Most of these deaths occur in rural areas of South and East Asia and many are preventable by simple measures such as legislative bans on the import and sale of the most toxic pesticides [2]. The benefits of restricting toxic pesticides on the incidence of suicide by pesticide poisoning have been shown in some countries [3,4] and such restrictions do not appear to impact on agricultural yield [5].

Previous studies have suggested that geographic variations in access to particular suicide methods may influence regional suicide rates. For example, it has been shown that stricter firearm restrictions [6] and lower levels of household firearm ownership [7] are associated with lower regional firearm suicide rates in the US. In Australia, areas with fewer vehicles built prior to the country's stringent carbon monoxide emission laws had lower rates of motor vehicle exhaust gas suicide [8]. In contrast, the geographic distribution of pesticide suicides in relation to markers of access to pesticides has received little attention in the research literature, although previous studies have documented substantial area variations in pesticide poisoning mortality in the US [9], Sri Lanka [10] and South Korea [11,12]. A study of Taiwan's 23 cities/counties showed that areas with a high proportion of resident agricultural workers had a high incidence of suicide by solids or liquids poisoning [13]; although the majority of these deaths were thought to be pesticide suicides the exact proportion was unknown. In contrast, a Sri Lankan study found no ecological association between the size of an area's agricultural population and its incidence of self-poisoning, although in areas where a high proportion of the population worked in agriculture pesticide poisonings were proportionately more frequent than in those areas with fewer agricultural workers [10]. Improved knowledge of geographic patterning of pesticide suicides may inform targeted prevention strategies.

The aim of this study was to investigate the contribution made by pesticide suicides to the geographic distribution and rural-urban differences of suicide in Taiwan, an island nation situated off the East coast of China with a population of approximately 23 million people. The ecological associations of pesticide suicide with area characteristics were also investigated. Throughout the paper the term 'pesticide' was used as a general term for a range of different products including insecticides, herbicides, fungicides and rodenticides.

## Methods

### Suicide and population data

Suicide data for people aged 15+ years were extracted from the Taiwanese national mortality data file for years 2002-2009. Suicide by pesticide poisoning was identified using the International Classification of Diseases, Tenth Revision (ICD-10) codes X68 (suicide by pesticide poisoning), Y18 (undetermined death by pesticide poisoning) and X48 (accident by pesticide poisoning), as the latter two cause-of-death categories contain probable 'missed' suicides in Taiwan [14]. Total suicides (all methods) were identified using the following ICD-10 codes: X60-X84 (suicide), Y10-Y34 (undetermined death), W75-W76 (accident by suffocation) and X48 (accident by pesticide poisoning) [14]. 'Non-pesticide' suicides were suicides by methods other than pesticide poisoning; these methods included i) poisoning using solid or liquid substances other than pesticides (X60-X65, X69, Y10-Y15, Y19); ii) poisoning using non-domestic gases (X67 and Y17); iii) hanging (X70, Y20, W75-W76); iv) drowning (X71 and Y21); v) jumping (X80 and Y30); and vi) all other methods. Each suicide was assigned to one of Taiwan's 358 administrative districts (median population aged 15+ years = 27 000) according to the individual's registered residence as recorded on the death certificate. Sensitivity analyses were conducted by restricting the analyses to certified suicides only (X60-X84).

Population data were obtained from the Demographic Fact Books (2002-2009) published by Taiwan's Ministry of the Interior (http://www.moi.gov.tw/stat/year.aspx). These provide annual population statistics for each district, based on the census and the national household registration system.

### Rural-urban definition

An urbanization index developed by the National Health Research Institute, Taiwan [15], was used to categorize each district into one of seven categories from level 1 (most urban) to level 7 (most rural). The index was based on five indicators drawn from the national census and Taiwan's register of physicians for the year 2000: i) population density, ii) population with college or higher educations, iii) population aged 65 or over, iv) population working in agriculture and v) the density of physicians. The average population (aged 15 years or above) of each of the seven categories of urbanicity are given in Table 1. Altogether 126 of Taiwan's 358 districts were categorised as urban (levels 1-3; 72% of the population) and 232 as rural (levels 4-7; 28% of the population); this is in keeping with official statistics indicating that 77% of residents lived in urban areas in 2000 [16].

**Table 1:** Numbers, percents and rates of pesticide and non-pesticide suicides by urbanization level in Taiwan, 2002-2009

	Population aged 15+ (N = 18 457 000)		Pesticide suicide (N = 4913)			Non-pesticide suicide (N = 31 197)		
**Urbanization level**	**n**	**(%)**	**n**	**(%)**	**Rate per 100 000**	**n**	**(%)**	**Rate per 100 000**

1 (most urban)	4 160 000	(22.5)	183	(3.7)	0.5	7138	(22.9)	20.7

2	5 417 000	(29.3)	712	(14.5)	1.6	9442	(30.3)	21.1

3	3 748 000	(20.3)	820	(16.7)	2.9	6554	(21.0)	21.8

4	2 936 000	(15.9)	1377	(28.0)	5.4	4746	(15.2)	19.3

5	470 000	(2.5)	308	(6.3)	6.7	749	(2.4)	18.2

6	881 000	(4.8)	885	(18.0)	10.9	1323	(4.2)	17.6

7 (most rural)	845 000	(4.6)	628	(12.8)	8.7	1245	(4.0)	17.6

### Area characteristics

To investigate the associations between the proportion of people working in farming in a particular area and the incidence of pesticide suicide in that area, data for the proportion of workers involved in agriculture in each district were obtained from the most recent (2000) census [17]. In the main multivariable analyses we assessed the possible confounding effects of two area factors: the proportion of lone-parent households (an indicator of social fragmentation) and median household income (an indicator of poverty) because a recent study showed that these factors are strongly associated with small-area variations in overall suicide rates in Taiwan [18]. In a sensitivity analysis we assessed the effect of controlling for additional variables associated with area suicide rates [18] - population mobility (i.e. the proportion of people who moved in or moved out from the district during 2000) and the proportion of divorced/separated population (also an indicator of social fragmentation) - as they both showed weak associations with area suicide rates in our previous analysis. In a separate sensitivity analysis we additionally controlled for area population density, which is also strongly associated with area suicide rates in Taiwan [18]; however, it should be noted that this variable was highly correlated with the proportion of people working in agriculture (r = -0.73) rendering interpretation of independent associations of population density/the size of the agricultural workforce problematic due to collinearity [19]. Data on lone-parent households and divorced/separated population for 2000 were extracted from the census [17] and those of median household income were from the Income Tax Statistics [20]. Data for population mobility and population density were extracted from the Taiwanese Demographic Fact Books (2000).

Although suicide data were from 2002-2009, rural-urban definitions and area characteristics were based on data from 2000 because that was the most recent census information available at the time of the study. All data used in the study are publicly available but access to the suicide and census data is subject to review by the Taiwanese government. This study used officially published data and therefore ethical approval is not required.

### Statistical analysis

'Raw' (unsmoothed) standardized mortality ratios (SMRs), i.e. the ratio of the observed to expected number of pesticide suicides, were calculated for each of the 358 administrative districts for those aged 15+ years. The 'expected' number of suicides in each area was estimated by multiplying the national sex-age-specific suicide rates (in 5-year age-bands) and the corresponding sex-age-specific population in each district. Data over the 8-year period (2002-2009) were aggregated to ensure sufficient events in each area. However, SMR estimates can still be unstable in small areas/populations as suicide is a relatively rare outcome (annual incidence in Taiwan = 24.5 per 100 000 per year) - a small change in the number of suicides may have a marked impact on the SMR estimates [21]. To address this issue Bayesian hierarchical models were used to estimate the 'smoothed' SMR for each district. The 'smoothed' estimates are the weighted averages of the SMRs for the area of interest and the mean risk in the neighboring areas or in the whole region studied, with the weights depending on the variability observed among areas of the study region. In this way, smoothed estimates of SMRs for each area can 'borrow strength' from data in the neighboring areas which share similar characteristics or the whole region being studied [22]. This improves the precision of the SMR estimates and takes account of small area variations in rates, thus enabling an investigation into the real differences in suicide risk between geographic areas.

The Bayesian hierarchical models were based on a Poisson assumption for the observed number of suicides, with random effects allowing for non-structural variability (heterogeneity across all areas in the study region) and structural variability (autocorrelation between neighboring areas) [21,23]. Sets of districts that share a border were defined as neighboring areas. Bayesian hierarchical models were estimated using the Markov chain Monte Carlo methods implemented in WinBUGS version 1.4. Vague prior distributions were used, and convergence of the simulations was assessed using the Gelman-Rubin statistic [24], based on three parallel chains. The built-in conditional autoregressive (CAR) distribution in WinBUGS was used to specify the spatially structural variability [25]. Hierarchical models were also constructed to investigate the associations of area characteristics with suicide both before and after controlling for all other characteristics. All analyses used standardized levels (z scores) of area characteristics or their log-transformed values when the distributions of raw values were positively skewed.

Method-specific age-standardized suicide rates were calculated for rural and urban areas, as well as areas in the seven urbanization levels, based on the World Health Organization (WHO) world standard population [26].

### Mapping

Choropleth maps, with different shades of color representing different incidence rates, were used to represent the geographic patterning of suicide. A divergent red-blue color scheme was used [27]; with varying shades used for areas with high (red) and low (blue) suicide rates; the central (average) category was shaded white. Maps were produced using ArcGIS Version 9.3.

Unsmoothed and smoothed SMRs for each district were ranked into eight categories; each of these was assigned a different color/shade as described above. Since the distribution of SMRs was skewed, symmetric categories on the logarithmic scale centered around 1.00 (the global mean of the study region) were preferred to dividing districts equally into quartiles/quintiles etc., as such categorization is sensitive to the distribution of data and may group values that differ greatly into broad and heterogeneous bands. The central category was chosen to include a range spanning 0.5 standard deviation below one to 0.5 standard deviation above one (i.e. SMRs of 0.9-1.1). Second, the most extreme categories at two ends of the distribution represented rate ratios that were a half or a double of the national average (i.e. < 0.5 and > 2.0 respectively). Last, the intermediate two breaks between the middle and the most extreme categories were separated at values that were two standard deviations away from one (i.e. 0.5-0.67 and 0.67-0.9 below one, and 1.1-1.5 and 1.5-2.0 above one). These resulted in seven categories that are symmetrical on the logarithmic scale (< 0.5, 0.5-0.67, 0.67-0.9, 0.9-1.1, 1.1-1.5, 1.5-2.0 and 2.0-5.0); one additional category was used to represent the highest values (> 5.0).

## Results

### Spatial patterning of suicide

Taiwan's urban region includes most of the western part of the island, with four major cities - Taipei in the north, Taichung in middle Taiwan, and Tainan and Kaohsiung in the southwest; population aged 15+ in these four cities was 2 176 000, 815 000, 617 000 and 1 244 000 respectively. Hualien (population aged 15+ = 87 000) is the only city in East Taiwan, which otherwise comprises mostly rural and mountainous areas.

Between 2002 and 2009 there were 36 110 suicides in Taiwan; 4913 (13.6%) of these were pesticide poisonings. 3950 (80.4%) of the pesticide deaths were certified as suicides, 639 (13.0%) as undetermined deaths and 324 (6.6%) as accidents. The proportion of males among the pesticide suicides (71.7%) was similar to that for non-pesticide suicides (68.2%), though pesticide suicides tended to be older (mean age 55.1 years) than non-pesticide suicides (mean age 47.7).

In 2002-2009 pesticide poisoning was the third most commonly used method in Taiwan, following hanging (30.2%) and poisoning using non-domestic gases (25.0%); pesticide poisoning accounted for 69% of all suicides from poisoning using solid or liquid substances. Table 2 summarizes the distributions of pesticide, non-pesticide and overall suicides and the area characteristics investigated across the 358 Taiwanese districts. Raw (unsmoothed) SMRs for pesticide suicides showed a striking 65-fold difference (90% range 0.08-5.18) even when the most extreme 10% values were excluded. There was also a marked 22-fold difference in smoothed SMRs (90% range 0.20-4.49). In contrast, non-pesticide suicides showed much less spatial variation; unsmoothed and smoothed SMRs showed 2.5-fold (90% range 0.55-1.40) and 1.6-fold (90% range 0.78-1.21) differences respectively.

**Table 2:** Number of suicides, raw standardized mortality ratios (SMRs), smoothed SMRs and area characteristics across Taiwan's 358 districts

	Mean (standard deviation)	Median (5th centile, 95th centile)
Pesticide suicide^a ^(2002-2009)		

Number of suicides	13.7 (10.4)	11.5 (1, 35)

Raw SMRs	1.88 (1.83)	1.47 (0.08, 5.18)

Smoothed SMRs	1.80 (1.45)	1.47 (0.20, 4.49)

Non-pesticide suicide^b ^(2002-2009)		

Number of suicides	87.1 (111.1)	45 (6, 310)

Raw SMRs	0.95 (0.27)	0.95 (0.55, 1.40)

Smoothed SMRs	0.96 (0.15)	0.93 (0.78, 1.21)

Overall suicide^c ^(2002-2009)		

Number of suicides	100.9 (113.7)	59 (9, 318)

Raw SMRs	1.08 (0.34)	1.04 (0.69, 1.59)

Smoothed SMRs	1.06 (0.17)	1.04 (0.82, 1.37)

Area characteristics (2000)		

Agricultural workforce (%)^d^	19.6 (16.4)	15.8 (0.6, 50.0)

Lone-parent households (%)^d^	5.9 (1.3)	5.7 (4.2, 8.5)

Median household income (1000 Taiwan dollar)^e^	512.7 (78.0)	498.0 (415.0, 650.0)

^a^Including certified suicides, undetermined deaths and accidents from pesticide poisoning

^b^Including certified suicides, undetermined deaths and accidents from suffocation

^c^Including certified suicides, undetermined deaths and accidents from pesticide poisoning/suffocation

^d^Data from the 2000 census

^e^Data from the 2000 Income Tax Files

As shown in Table 2, the mean and median of raw and smoothed SMRs for pesticide suicides were higher than one (i.e. the national average); this was because a large number of districts (mostly rural) with small populations showed higher than the national average rates while a smaller number of districts that were urban and had much larger populations showed lower than the national average rates.

**Figure 1:**
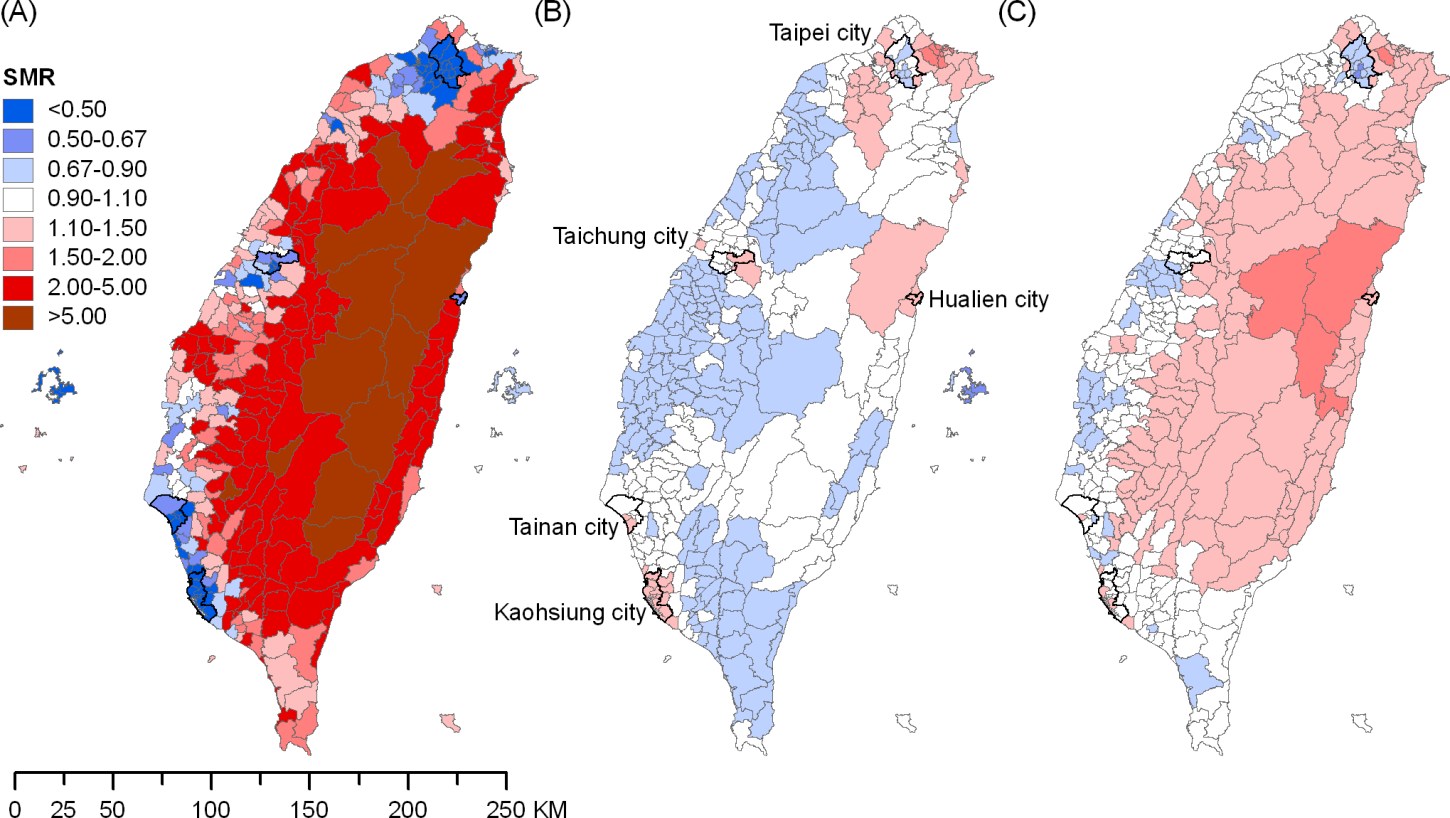
**Maps^a ^for (A) pesticide, (B) non-pesticide and (C) overall suicide^b ^across Taiwan's 358 districts, 2002-2009**. ^a^Smoothed standardized mortality ratios (SMRs) were mapped here. ^b^Including certified suicide, undetermined death, and accident from pesticide poisoning and suffocation.

Figure 1 shows the geographic distributions of smoothed SMRs for (A) pesticide suicide, (B) non-pesticide suicide and (C) overall suicide. The patterns for unsmoothed SMRs were similar but somewhat less clear due to unstable estimates in sparsely populated areas (data not shown). Higher rates of pesticide suicide were found in East and Central Taiwan, with a concentration of the highest rates in the most rural areas (Figure 1A). One hundred and thirty (36.6%) districts had a SMR above two; they accounted for nearly half (46.6%) of all pesticide suicides, but only 13.4% of Taiwan's population aged 15+ live in these areas. In contrast, five major cities showed the lowest rates; they covered 26.8% of overall population but accounted for only 6% of pesticide suicides.

In contrast to the distinct geographic pattern of pesticide suicide, there was no strong spatial patterning for non-pesticide suicide, although above average rates were found in Kaohsiung city and some areas surrounding Taipei city (Figure 1B). The geographic distribution of overall suicides (Figure 1C) was more similar to that of pesticide suicides than non-pesticide suicides, indicating the impact of pesticide suicide on the overall spatial patterning of suicide in Taiwan.

In the sensitivity analyses including certified suicides only, the main features of the spatial patterning of pesticide, non-pesticide and overall suicides were similar to those based on data for certified and possible suicides combined (data not shown).

### Rural-urban differences

Overall suicide rates were higher in rural than urban areas (25.6 versus 22.8 per 100 000) in Taiwan. Table 3 shows rural and urban rates of suicide and their differences by suicide method. The differences in overall suicide rates between rural and urban areas were mostly attributable to pesticide suicide - the rural-urban difference in pesticide suicide rates was 5.4 per 100 000 (7.0 versus 1.6 per 100 000), while for other methods differences ranged from 0.4 per 100 000 for hanging (7.4 versus 7.0 per 100 000) to -1.4 per 100 000 for jumping (1.6 versus 3.0 per 100 000). There was a substantial gradient in pesticide suicide rates across the seven categories of urbanization - rates were 0.5, 1.6, 2.9, 5.4, 6.7, 10.9 and 8.7 per 100 000 for areas in level 1 (most urban) to 7 (most rural) respectively (Table 1). Nearly two thirds of pesticide suicides (65.1%) were in rural areas (levels 4-7), yet these areas included only 28% of Taiwan's population aged 15+ years. In contrast, non-pesticide suicide rates showed much less variations - they ranged from 21.8 (level 3) to 17.6 (level 7) per 100 000 (Table 1).

**Table 3:** Age-standardized suicide rates per 100 000 in rural and urban areas in Taiwan, 2002-2009

*Suicide method*	Rural areas (rate per 100 000)	Urban areas (rate per 100 000)	Rural-urban difference (rate per 100 000)
Pesticide suicide	7.0	1.6	5.4

Non-pesticide suicide	18.6	21.2	-2.6

Poisoning using solid/liquids other than pesticides	1.5	1.4	0.1

Poisoning using non-domestic gases	5.0	6.3	-1.3

Hanging	7.4	7.0	0.4

Drowning	1.5	1.8	-0.2

Jumping	1.6	3.0	-1.4

Other methods	1.6	1.7	-0.1

### Ecological analyses

**Figure 2:**
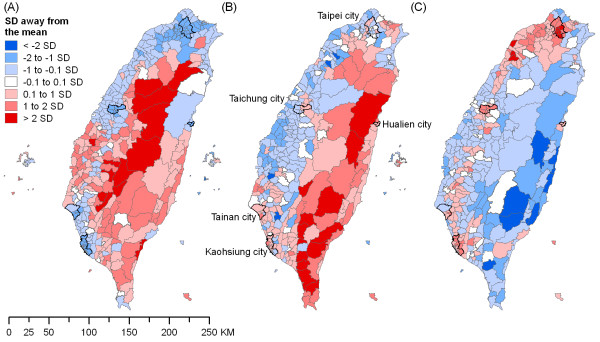
**Maps^a ^for (A) agricultural workforce (%), (B) lone-parent households (%) and (C) median household income**. ^a^According to standard deviation (SD) away from the national mean, across Taiwan's 358 districts, 2000.

Ecological analyses showed that a district's pesticide suicide rate was strongly associated with the proportion of the workforce involved in agriculture (Table 4). Figure 2 shows the maps for the proportions of workforce involved in agriculture, lone-parent households and median household income across Taiwan's 358 districts in 2000. In the unadjusted model, one standard deviation increase in the proportion of workforce in agriculture was associated with a 78% increase in district rates of pesticide suicide (rate ratio [RR] = 1.78, 95% Credible Interval [CrI] 1.63-1.94), although the strength of association decreased after controlling for an area's levels of lone-parent households and median household income (RR = 1.58, 95% CrI 1.44-1.74). When additionally controlling for area levels of divorced/separated population and population mobility in a sensitivity analysis, the strength of the association further decreased (RR = 1.46, 95% CrI 1.31-1.61). In another sensitivity analysis that additionally controlled for an area's level of population density, the association of pesticide suicide rates with the proportions of workforce involved in agriculture attenuated markedly (RR = 1.24, 95% CrI 1.10-1.38), but this may be an underestimate as population density was strongly correlated with an area's level of agricultural workforce (Pearson correlation coefficient = -0.73) and likely to be a partial indicator of agricultural workforce and exposure to pesticides in itself.

**Table 4:** Rate ratios (and 95% Credible Intervals) of suicide per SD^a ^increase in three area characteristics

Area characteristics	Rate ratio (95% Credible Intervals), unadjusted	Rate ratio (95% Credible Intervals), adjusted for all other characteristics
Pesticide suicide^b^		

Agricultural workforce (%)	1.78 (1.63, 1.94)	1.58 (1.44, 1.74)

Lone-parent households (%)^e^	1.06 (0.97, 1.17)	1.09 (1.10, 1.17)

Median household income (1000 Taiwan Dollar)^e^	0.63 (0.58, 0.68)	0.75 (0.69, 0.82)

Non-pesticide suicide^c^		

Agricultural workforce (%)	0.93 (0.90, 0.96)	0.91 (0.87, 0.95)

Lone-parent households (%)^e^	1.12 (1.08, 1.15)	1.10 (1.06, 1.13)

Median household income (1000 Taiwan Dollar)^e^	0.98 (0.96, 1.10)	0.95 (0.92, 0.98)

Overall suicide^d^		

Agricultural workforce (%)	1.09 (1.06, 1.13)	1.06 (1.03, 1.10)

Lone-parent households (%)^e^	1.10 (1.07, 1.13)	1.10 (1.07, 1.13)

Median household income (1000 Taiwan Dollar)^e^	0.90 (0.88, 0.92)	0.94 (0.91, 0.96)

^a^Standard deviation (SDs for agricultural workforce, lone-parent households and median household income were 16.4%, 1.3% and 78,000 Taiwan dollar [= 2500 US dollar] respectively)

^b^Including certified suicides, undetermined deaths and accidents from pesticide poisoning

^c^Including certified suicides, undetermined deaths and accidents from suffocation

^d^Including certified suicides, undetermined deaths and accidents from pesticide poisoning/suffocation

^e^Values were log-transformed before standardization due to their skewed distributions

In contrast, rates of non-pesticide suicide were lower in districts with a higher proportion of workforce involved in agriculture (unadjusted RR = 0.93, 95% CrI 0.90-0.96; adjusted RR = 0.91, 95% CrI 0.87-0.95). Overall suicide rates were positively associated with an area's level of agricultural workforce (unadjusted RR = 1.09, 95% CrI 1.06-1.13; adjusted RR = 1.06, 95% CrI 1.03-1.10), although the strengths of associations were much weaker than those for pesticide suicide.

In the sensitivity analyses including certified suicides only, results were similar to those based on certified and possible suicides combined (detailed data not shown). In the models controlling for lone-parent households and median household income, districts where a higher proportion of population worked in agriculture had higher rates of pesticide suicide (adjusted RR = 1.76, 95% CrI 1.48-2.09), lower rates of non-pesticide suicide (adjusted RR = 0.92, 95% CrI 0.87-0.99) and higher overall suicide rates (adjusted RR = 1.09, 95% CrI 1.03-1.16).

## Discussion

There were striking geographic variations and a distinct spatial patterning for pesticide suicide in Taiwan, with higher rates in Central and East Taiwan and lower rates in major cities. The highest rates of pesticide suicide were found in the most rural areas. Areas where rates were more than two times higher than the national average accounted for almost half (46.6%) of all pesticide suicides but were inhabited by only 13.4% of the population aged 15+. The geographic distribution of overall suicides was more similar to that of pesticide suicides than non-pesticide suicides. Rural-urban differences in suicide were mostly due to pesticide suicide. Areas where a higher proportion of the population worked in agriculture had higher pesticide-specific and overall suicide rates but not non-pesticide suicide rates. The association of the proportion of agricultural workforce with area pesticide suicide rates persisted after controlling for area indicators of socioeconomic disadvantage, which may themselves increase the risk of suicide.

### Strengths and limitations

To our knowledge this is the first nationwide analysis of small-area variations in suicide from pesticide poisoning. The availability of small-area data allowed a detailed investigation of the spatial distribution of pesticide suicide. The study has a number of limitations. First, data for non-fatal self-poisoning using pesticides were unavailable. This study was unable to determine whether the spatial patterning of pesticide suicide was due to variations in the incidence of self-poisoning or in case fatality following pesticide poisoning; area differences in case-fatality could be caused by area differences in access to medical services or variations in the toxicity of the pesticides commonly taken in episodes of self-poisoning (due to variations in agricultural practices). Second, an area's proportion of workforce in agriculture was used as the proxy indicator of access to pesticides; other variables may be better markers of pesticide accessibility, e.g. pesticide sales, for which small-area data were unavailable. Third, we aggregated data for 8 years (2002-2009) to ensure a sufficient number of events in small areas. Such aggregation may mask area-specific changes in suicide rates over this period; analysis of relevant data revealed that although pesticide suicide rates did decline over this period, the reduction was similar in rural and urban areas (32% and 33% respectively). Last, there are geographic variations in the quality of suicide statistics in Taiwan [28]; however, results were similar when using data for certified suicides only or certified and possible suicides combined.

### Comparison with previous studies

The study showed substantial geographic variation in the incidence of pesticide suicide and its impact on the overall spatial patterning of suicide in Taiwan. In contrast, non-pesticide suicides showed no strong spatial patterning. A recent study from Taiwan showed that self-poisoning using solid or liquid substances demonstrated the greatest geographic variations compared with other common suicide methods [18]; the present study showed that 69% of such solids/liquids poisoning suicides were due to pesticides. This study also showed that pesticide suicide contributed to most of the rural-urban disparity of suicide in Taiwan. A recent study demonstrated that such disparity had attenuated over the last decade after the emergence of charcoal-burning suicides around 1998-2000, which concentrated in mostly urban areas [29].

Areas with a high proportion of their labour force working in agriculture experienced higher rates of pesticide suicide, but not non-pesticide suicide, both before and after controlling for area socioeconomic disadvantage. The association suggested the role of means availability in the spatial distribution of pesticide suicides - suicide rates were higher in areas where there was easier access to pesticides, indicated by a higher proportion of people working in agriculture. Likewise, an ecological analysis in South Korea found strong association of pesticide self-poisoning mortality rates with an index of farming activities and population [12]. In contrast, a recent study in southern rural Sri Lanka found that areas with a high proportion of population working in agriculture had low rates of self-poisoning, although a greater proportion of episodes in these areas involved pesticides [10]. However, this study was limited to investigating only a relatively small, mainly agricultural area in southern Sri Lanka. The socioeconomic characteristics of workers in this area are likely to be quite different from those in Taiwan and South Korea. Furthermore, farmers in different countries may be involved in different types of cultivation with varying requirements of pesticide application, and thus the 'population working in agriculture' may be a good indicator of pesticide accessibility in some but not other countries/regions. Recent person-based studies in rural China provided further evidence for pesticide access as an independent risk factor for suicide in residents controlling for individuals' socioeconomic conditions and mental disorders [30].

### Public health implications

The WHO acknowledges that pesticide ingestion is among the mostly frequently used methods of suicide worldwide [31]. Pesticides are readily available in rural areas of developing countries and commonly used in impulsive acts of self-poisoning following acute life crisis [32,33]. This leads to many tragic deaths due to the high case fatality associated with pesticide ingestion [34]. One strategy to reduce these deaths is to restrict the availability of toxic pesticides. Studies in countries such as Finland [3] and Sri Lanka [4] showed marked reductions in pesticide suicides after restricting their use. In keeping with these, the present study showed that the spatial distribution of pesticide suicide was strongly associated with the distribution of agricultural workforce, an indicator of pesticide availability/accessibility. Other strategies for preventing pesticide suicides included safe storage in lockable boxes [35,36], centralized communal storage, education to users, retailers and community leaders [37], stopped using all pesticides by adopting non-pesticide management policy [38] and improved medical treatment of pesticide poisoning [39]. A study of the effectiveness of locked boxes is currently underway in Sri Lanka [40]. It is not yet clear which intervention is the most promising [41] because no comparative cost-effectiveness analysis is possible at the moment. A hazard reduction approach suggests that banning the more toxic pesticides from agricultural practice would be most effective [42]. However, the agricultural costs and the healthcare benefits of switching to less toxic pesticides are not currently known.

This study showed that, in Taiwan, some areas had disproportionately high numbers of pesticide suicides. In the eastern mountainous part and central rural areas of Taiwan, where rates of pesticide suicide were high, strategies such as restricting access to the highly toxic pesticides and improving the medical care of self-poisoning may reduce suicide mortality. Such prevention strategies may help to tackle inequality in suicide burden in Taiwan as pesticide suicide was the major contributor to geographic variations in overall suicide rates. Identification of areas with high rates of pesticide suicide will inform the allocation of prevention resources targeted at reducing these deaths.

## Conclusions

The spatial variation and patterning of pesticide suicide in Taiwan was striking, with around half of such deaths occurring in areas where only one eighth of the population live. Suicides by pesticide poisoning contributed to most of the geographic distribution and rural-urban difference in overall suicides, compared with suicides by other methods. Easy access to pesticides, indicated by the size of the agricultural workforce in an area, was associated with a high incidence of pesticide suicide but not non-pesticide suicides, after accounting for markers of area socioeconomic disadvantage. The results highlighted the potential benefits of targeted suicide prevention strategies such as restricting access to highly toxic pesticides.

## Competing interests

The authors declare that they have no competing interests.

## Authors' contributions

S-SC, T-HL and DG contributed to study design. S-SC undertook the analysis with input from JS and DG. All authors contributed to the interpretation of results. S-SC wrote the first draft of the paper. All authors have read and approved the final manuscript.

## Pre-publication history

The pre-publication history for this paper can be accessed here:

http://www.biomedcentral.com/1471-2458/12/260/prepub
